# Supplementary material for: Mechanism and cellular function of direct membrane binding by the ESCRT and ERES-associated Ca2+-sensor ALG-2
Source: Proc Natl Acad Sci U S A. 2024 Feb 22;121(9):e2318046121. doi: 10.1073/pnas.2318046121 (PMC10907313; doi:10.1073/pnas.2318046121)
Supplement: Supplementary file 1 — Appendix 01 (PDF) [file pnas.2318046121.sapp.pdf]

Supplementary Information for

Mechanism and cellular function of direct membrane binding by the ESCRT and ERES-associated Ca^{2+}-sensor ALG-2

Sankalp Shukla[1, 2], Wei Chen[3], Shanlin Rao[4], Serim Yang[1], Chenxi Ou[1, 2], Kevin P. Larsen[1,2], Gerhard Hummer[4,5], Phyllis I. Hanson[3], and James H. Hurley[1, 2, 6,*]

Correspondence: James H. Hurley
Email: jimhurley@berkeley.edu

This PDF file includes:

Figure S1 to S5

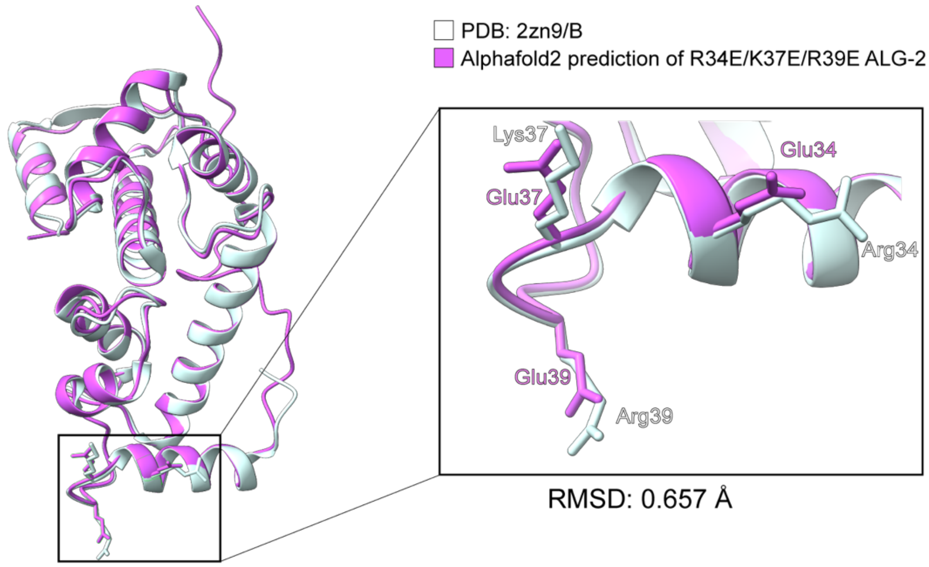

Fig. S1. AlphaFold comparison between WT and R34E/K37E/R39E ALG-2 mutant. Overlay of the crystal structure of des3-20 WT ALG-2 (PDB - 2zn9: B) (white) with AlphaFold2 (AF2)–predicted model for R34E/K37E/R39E ALG-2 (magenta). The C_α comparison between residues 24 to 188 (which are ordered) between the des3-20 WT ALG-2 (PDB - 2zn9: B) and AF2–predicted model R34E/K37E/R39E ALG-2 mutant, resulted in a RMSD value of 0.657Å and a TM-Align score of 0.978 (when normalized by length of des3-20 WT ALG-2 (PDB - 2zn9: B)). RMSD, root mean square deviation. TM-score, template modeling score.

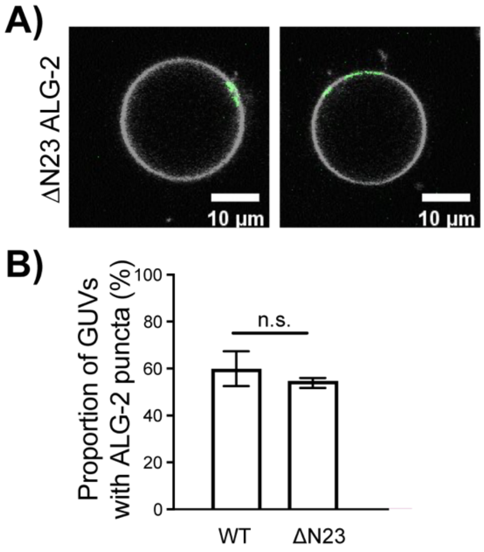

Fig. S2. Membrane binding of N-terminally deleted ALG-2. The ΔN23 ALG-2 fluorescently labeled with Atto 488 was incubated with 30% DOPS containing GUVs. (A) ΔN23 ALG-2 (green) was recruited to the 30% DOPS GUVs (white). The images are depicted as a merged channels between the ALG-2 and membrane channel. (B) The proportion of GUVs that had at least one ΔN23 ALG-2 punctum (green) on their periphery (white) were plotted for fluorescently labeled WT (n = 1388 GUVs) and ΔN23 ALG-2 (n = 603 GUVs). The data are shown as mean ± SD (vertical line). All results are from at least three independent experiments.

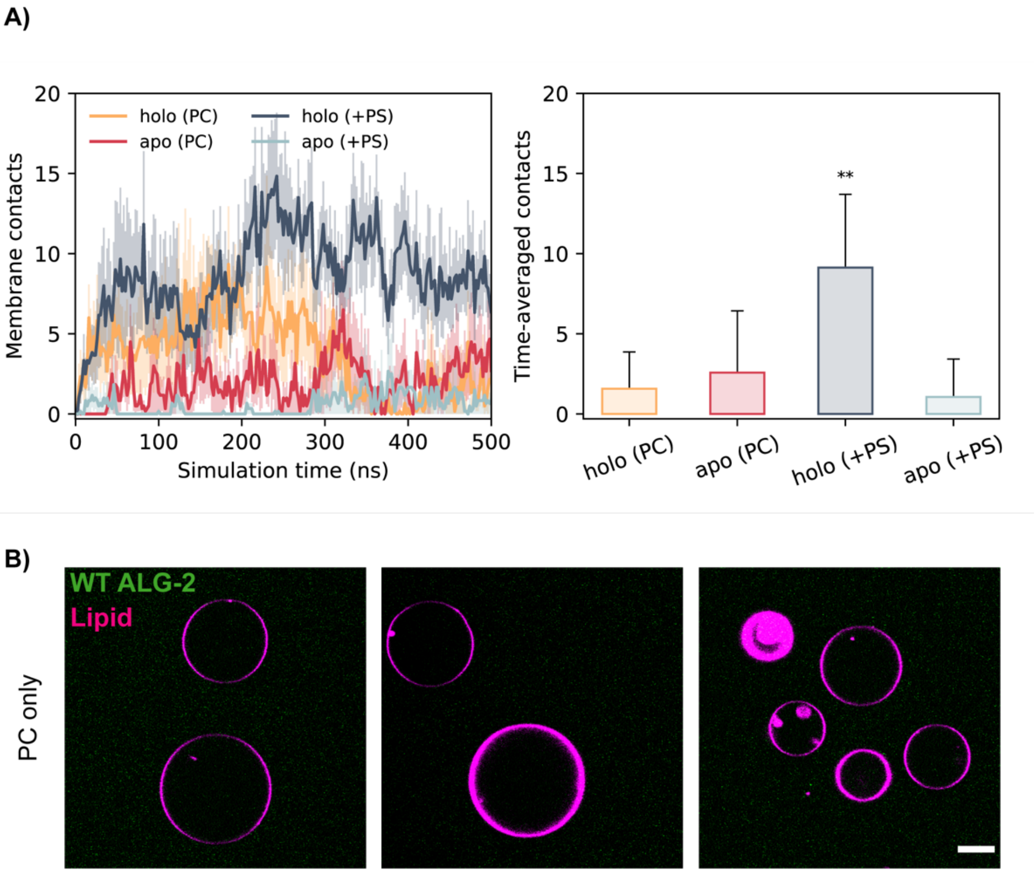

Fig. S3. Interaction of WT ALG-2 with PC only and PC/PS membrane. A) Molecular dynamics simulations with PC only and PC/PS membranes. Number of ALG-2 residues forming membrane contacts during all-atom molecular dynamics simulation replicates, comparing between the Ca^{2+}-bound (holo) and apo forms of ALG-2 binding to PC membranes and to membranes containing 30% PS. The mean (solid lines) and standard errors (semi-transparent shading) are plotted over time for six simulation replicates. Time-averaged membrane contacts and standard deviations are also calculated between t = 300 ns and 500 ns of simulation replicates. A statistically significant ($0.001 < p < 0.01$; one-tailed Student's t-test) difference in binding, compared with the holo protein docking to PC membranes, is denoted by an asterisk. B) Atto 488 labeled WT ALG-2 (200 nM) was incubated with DOPC only GUVs (99.5% DOPC, 0.5% DOPE-Atto 647). No recruitment of WT ALG-2 to PC membranes was observed. The scale bar is 10 µm.

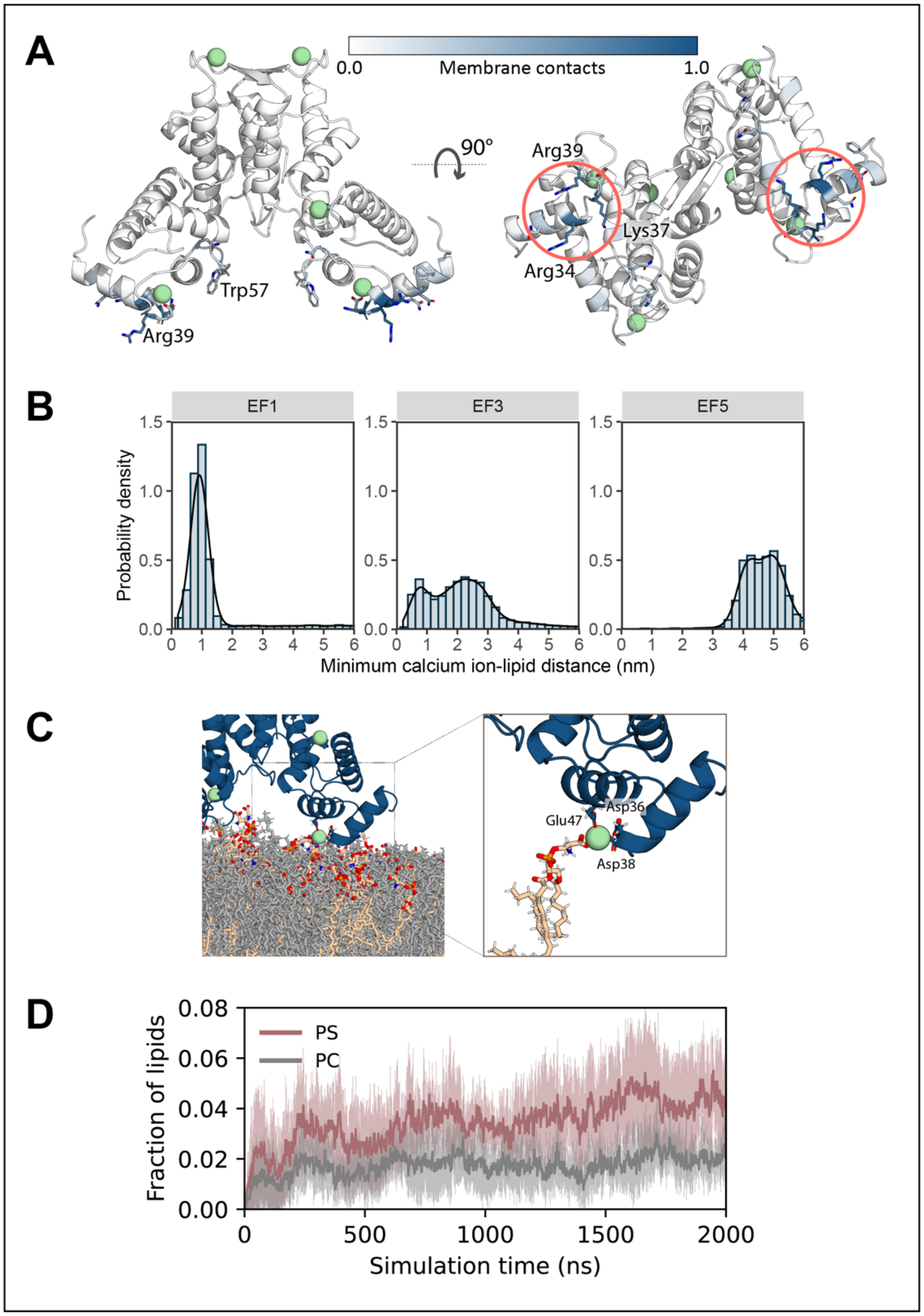

Fig. S4. Molecular dynamics simulations of ALG-2 membrane interactions. (A) Structure of Ca^{2+}-bound ALG-2 (PDB ID: 2ZN9) with residues colored by their mean frequency of membrane contacts during the final 1 µs of each of six 2 µs simulation replicates (white to blue at increasing contact frequency). (B) Distribution of distances between Ca^{2+} ions bound to EF1, EF3, and EF5 and their nearest lipid atom through six 2 µs simulation replicates. (C) Molecular dynamics simulation snapshot capturing direct Ca^{2+}

coordination by phosphate oxygen atoms of a membrane PS lipid. (D) Fractions of the number of membrane PC and PS lipids, respectively, that are in contact with ALG-2 over time. Showing the mean values across six simulation replicates, with the standard deviations indicated as shaded gray and pink bands.

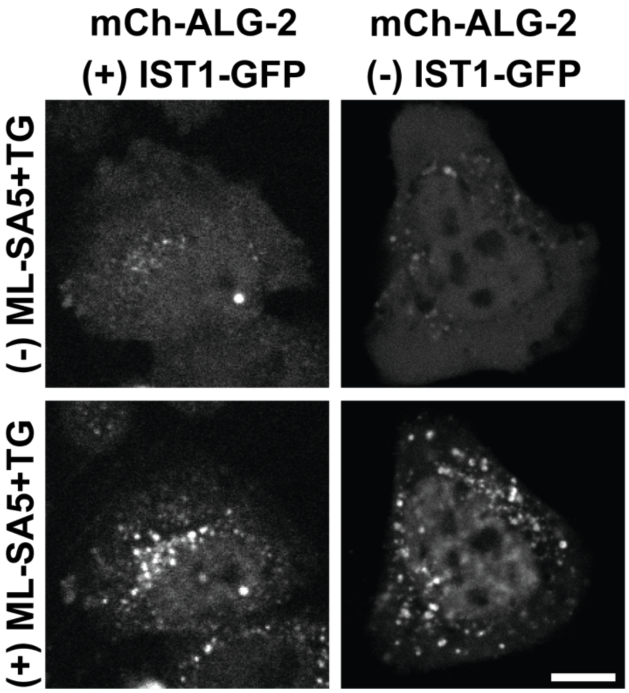

Fig. S5. ALG-2 overexpression in the absence of IST overexpression. ALG-2 KO HeLa cells were transfected with mCherry-ALG-2 alone or together with IST1-GFP. Cells were imaged live before and 15min after addition of ML-SA5 and TG. The scale bar is 10 μm.
